# 

**Published:** 1905-06

**Authors:** 


					﻿BUFFALO
MEDICALJOURNAL
1845 by AUSTIN E L/^TSkM Jx>*
’	'■	]' EDITOR:	\
WILLDAM WARREN POTTER, NTs®
-ASSISTANT EDITORS:
WILLIAM C. KRAUSS, M. D.
NELSON W. WILSON, M. D.
ASSOCIATE EDITORS:
JAMES WRIGHT PUTNAM, M. D.
JOHN PARMENTER, M. D.
HARVEY R. GAYLORD, M. D.
ERNEST WENDE, M. D.
JOHN A. MILLER, Ph. D.
MAUD J. FRYE, M. D.
For terms and other information relating to the Journal, see page vi.
				

